# Social cognitive outcomes are associated with improvements in mobility performance following lifestyle intervention in prostate cancer patients undergoing androgen deprivation therapy

**DOI:** 10.1371/journal.pone.0263136

**Published:** 2022-01-27

**Authors:** Zachary L. Chaplow, Alexander R. Lucas, Elizabeth Grainger, Christina Simpson, Ciaran M. Fairman, Victoria R. DeScenza, Jessica Bowman, Steven K. Clinton, Brian C. Focht

**Affiliations:** 1 Kinesiology, Department of Human Sciences, The Ohio State University, Columbus, Ohio, United States of America; 2 Department of Health Behavior and Policy, Virginia Commonwealth University, Richmond, Virginia, United States of America; 3 Division of Medical Oncology, Department of Internal Medicine, The Ohio State University, Columbus, Ohio, United States of America; 4 Comprehensive Cancer Center, The Ohio State University, Columbus, Ohio, United States of America; 5 Exercise Science Department, University of South Carolina, Columbia, South Carolina, United States of America; Pennington Biomedical Research Center, UNITED STATES

## Abstract

**Objective:**

To compare the effects of an exercise and dietary intervention with those of standard-of-care management upon change in lift and carry performance and mobility-related self-efficacy beliefs and explore associations in prostate cancer patients undergoing androgen deprivation therapy.

**Methods:**

32 prostate cancer patients (*M* age = 66.2 years; *SD* = 7.8) undergoing androgen deprivation therapy were randomly assigned to a 3-month exercise and dietary lifestyle intervention (*n* = 16) or standard-of-care management (*n* = 16). Outcome assessments were obtained at baseline, 2- and 3-month follow-up.

**Results:**

The lifestyle intervention resulted in significantly greater improvements in lift and carry performance (*p* = 0.01) at 2 Months (*d* = 1.01; p < 0.01) and 3 Months (*d* = 0.95; p < 0.01) and superior improvements in mobility-related self-efficacy at 2 Months (*d* = 0.38) and 3 Months (*d* = 0.58) relative to standard-of-care. Mobility-related self-efficacy (*r* = -.66; *p* = 0.006) and satisfaction with function (*r* = -.63; *p* = 0.01) were significantly correlated with lift and carry performance at 3 Months.

**Conclusions:**

The exercise and dietary lifestyle intervention yielded superior improvements in lift and carry performance and mobility-related self-efficacy relative to standard-of-care and key social cognitive outcomes were associated with more favorable mobility performance.

## Introduction

Androgen deprivation therapy (ADT) is the foundation of treatment of prostate cancer (PCa) and increasingly employed in effective multimodality therapy with men showing prolonged duration of therapy and survival. The adverse effects of ADT on PCa patients’ body composition and muscle strength increase risk of functional decline [1]. The concomitant loss of muscular strength and gain in fat mass observed with ADT have the potential to significantly compromise PCa patients’ capacity to perform basic mobility-related activities of daily living that require lifting and carrying objects such as laundry baskets or grocery bags. As difficulty with the physical demands of common daily activities is a primary predictor of mobility disability [2], countering declines in the ability to complete lift and carry tasks that accompany ADT is integral to the supportive care of PCa patients. In this regard, lifestyle interventions promoting physical fitness and healthy dietary intake consistently yield meaningful improvements in mobility performance among older adults [3,4] and recent findings underscore the potential utility of implementing lifestyle interventions for offsetting the deleterious effects of ADT in PCa patients [5,6].

Social Cognitive Theory [7] provides a theoretical framework for understanding the factors underlying adoption of lifestyle interventions for attenuating functional decline and delineates the potential role of key self-regulatory and motivational factors involved in exercise and dietary behavior change. For example, select social cognitive constructs, such as mobility-related self-efficacy (MRSE) judgments and satisfaction with function may have important behavioral and clinical implications for the utility of lifestyle interventions in preserving physical function and mobility among PCa patients undergoing ADT. MRSE is an established social cognitive construct reflecting one’s belief in their ability to successfully complete more challenging increments of a specific mobility-related physical task representative of a common activity of basic living (i.e., lift and carry task). Satisfaction with function represents one’s perceived satisfaction with their physical function and their subjective judgment of associated function-related outcome expectancies. Together, these constructs have been shown to predict functional decline [8], mediate the beneficial effects of lifestyle interventions upon mobility performance [5,9], and are key aspects of one’s agency to pursue goal-directed actions that are integral to successful adoption and maintenance of lifestyle behavior change in older adults with, or at risk, for mobility disability [10–12]. Consistent with these findings, results from our recently completed Individualized Diet and Exercise Adherence-Pilot (IDEA-P) trial demonstrate that a Social Cognitive Theory-based lifestyle exercise and dietary intervention yielded improvements in body composition, functional, and social cognitive outcomes relative to standard of care treatment in PCa patients on ADT [5,6,13,14].

As performance of basic functional tasks and mobility-related social cognitive beliefs are meaningful predictors of functional decline, the IDEA-P trial provides one of the first opportunities to examine the extent to which social cognitive outcomes are associated with lift and carry task performance following a theory-based lifestyle intervention among PCa patients undergoing ADT. Therefore, the purpose of this study is to conduct ancillary analyses of IDEA-P trial outcomes to: 1) examine change in lift and carry (L+C) performance and associated task specific MRSE beliefs following lifestyle (EX+D) and standard of care (SC) interventions; and 2) explore the post-intervention associations between L+C performance and select social cognitive outcomes. It was hypothesized that the EX+D intervention, relative to SC, would result in superior improvement in L+C performance and MRSE and that higher self-efficacy and satisfaction with function would be associated with more favorable post-intervention L+C performance.

## Methods

Detailed descriptions of the IDEA-P trial design and methods including eligibility criteria have been published previously [6,13,14]. However, a brief summary of the relevant procedures, interventions, and measures is provided here. The primary objectives of IDEA-P were (a) to determine the feasibility of delivering this specific EX+D intervention approach to prostate cancer patients on androgen-deprivation therapy; (b) to explore the preliminary efficacy of EX+D for improving clinically relevant functional, fitness, and patient-reported outcomes compared with SC treatment to inform the design of a subsequent large scale definitive efficacy trial; and (c) to examine the short-term adoption and maintenance of independent, self-regulated exercise and dietary behavior change for men undergoing androgen suppression therapy.

### Trials design

The IDEA-P trial was a single-blind, 2-arm randomized pilot trial with a 1:1 allocation ratio.

#### Participants and eligibility criteria, and settings

**Fig 1:**
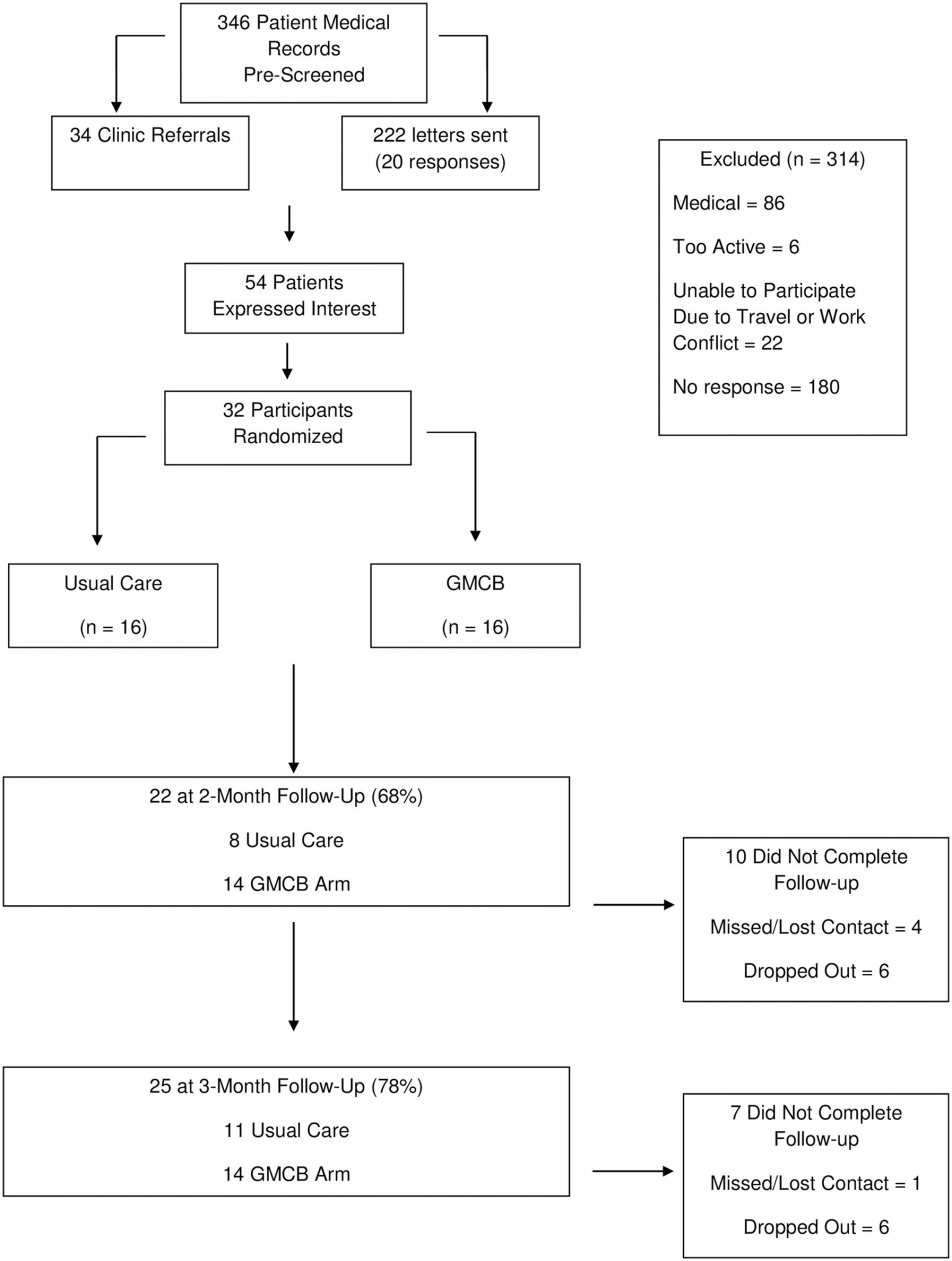
CONSORT diagram of participant flow through the IDEA-P trial.

A total of 32 prostate cancer patients on androgen-deprivation therapy were recruited to participate in the IDEA-P trial at The Ohio State University James Cancer Hospital and Comprehensive Cancer Center (Columbus, OH). Key eligibility criteria for the IDEA-P trial included: (a) histologically-defined diagnosis of prostate cancer based upon pathology reports and staging studies; (b) currently undergoing androgen-deprivation therapy with a planned course of at least 3 months of continuous therapy; (c) sedentary activity pattern with less than 60 min of structured exercise participation per week during the past 6 months, consistent with recent lifestyle intervention trial’s classification of inactivity; and (d) free of poorly controlled medical conditions that precluded safe participation in an exercise program. The Consolidated Standards of Reporting Trials (CONSORT) diagram demonstrating recruitment and retention of participants through the trial is depicted in Fig 1.

### Interventions

PCa patients enrolled in the IDEA-P trial were randomized into either the EX+D or SC intervention arms. The 3-month EX+D intervention was a Social Cognitive Theory-based lifestyle intervention that integrated exercise, diet, and group-mediated cognitive behavioral (GMCB) counseling in a tapered contact schedule to develop group support and mastery of self-regulatory skills facilitating independent exercise and healthier dietary intake. The personalized exercise component integrated one-hour supervised exercise sessions performed twice per week and involved a combination of resistance and aerobic exercise. Additionally, participants were encouraged to gradually progress towards accruing a total weekly volume of physical activity consistent with national guidelines for health and well-being [15]. GMCB counseling was delivered via small group (4–8 patients) sessions lasting 30 minutes in duration conducted immediately following center-based exercise sessions during months 1–2. The dietary component of the lifestyle intervention aimed to provide basic nutrition education/counseling to all participants, address contemporary topics in nutrition and cancer, and personalized guidance toward adopting changes in dietary intake consistent with current national nutritional objectives [16]. As reported previously [6], adherence to the supervised sessions during the EX+D intervention was 88%. The SC intervention involved standard treatment and disease management education, as well as complementary literature describing the WCRF/AICR dietary and physical activity guidelines [16]. To ensure equitable contact between treatment arms, 20-minute phone contacts, delivered by study staff, focusing on routine aspects of PCa self-management were conducted biweekly with men in the control arm. The trial was approved by The Ohio State University Institutional Review Board (Protocol # 2012C0008) and all participants completed informed consent prior to participation.

### Outcomes

The L+C test is a simulated activity of daily living task. Patients pick up a storage bin containing a 10lb weight from a shelf, carry the container 5m down a hallway, circling around a cone and walking back down the hallway to place the container back on the shelf. L+C performance was assessed as the total amount of time (in seconds) to complete the task [2]. The social cognitive constructs were assessed using the MRSE [3,13], and satisfaction with function [9] scales. The MRSE scale consisted of an 8-item scale involving hierarchically organized items assessing beliefs in successfully completing incrementally more challenging aspects of the L+C task on a response scale ranging from 0% (no confidence) to 100% (completely confident). The 9-item satisfaction with physical function and appearance (SWF) measure assessed patients on a 7-point scale ranging from -3 (Very Dissatisfied) to +3 (Very Satisfied). Each of the performance and self-report measures has well-established validity and reliability and have been used in prior randomized controlled lifestyle intervention trials [3,4,6,9,13]. All outcomes were assessed at baseline and 2 Month and 3 Month follow-ups.

### Sample size calculation

As noted in our prior trial publications [6,13,14], sample size was based on established recommendations for estimating sample size in pilot, randomized trials and adequate to obtain effect size estimates necessary to accurately set parameters for a subsequent, optimally-powered randomized controlled trial.

### Randomization

PCa patients were randomly assigned with equal probability to each of the 2 treatment arms using a 1:1 ratio following the completion of the baseline screening visit. The computer-generated randomization allocation sequence was sequentially numbered and sealed in opaque envelopes. The randomization allocation sequence was also concealed from study staff responsible for recruiting patients & conducting the baseline assessments.

### Blinding

As IDEA-P was a single-blind trial, all outcomes were assessed at baseline and 2 Month and 3 Month follow-ups by study staff that were blinded to intervention arm assignment.

### Statistical analysis

Analysis was conducted with separate 2 (Treatment: EX+D and SC) x 2 (Time: 2 Month and 3 Month) analyses of covariance (ANCOVA) with time on ADT and baseline values of each measure included in the models as covariates. All analyses were conducted using the intention-to-treat principle to account for missing data and the last-observation-carried-forward approach, used to impute change across time as zero. Statistical tests were two-tailed with an alpha level of 0.05 required for statistical significance. All analyses were conducted using SPSS 23.0 (IBM SPSS Statistics for Windows, Version 23, Armonk, NY; IBM Corp.). Additionally, Cohen’s *d* effect sizes were calculated to determine the magnitude of adjusted means differences and partial correlation analyses (Pearson correlation coefficient) controlling for time on ADT and baseline outcome values were conducted to examine the relationship between the social cognitive constructs and L+C performance at the 3 Month follow-up assessment.

## Results

**Fig 2:**
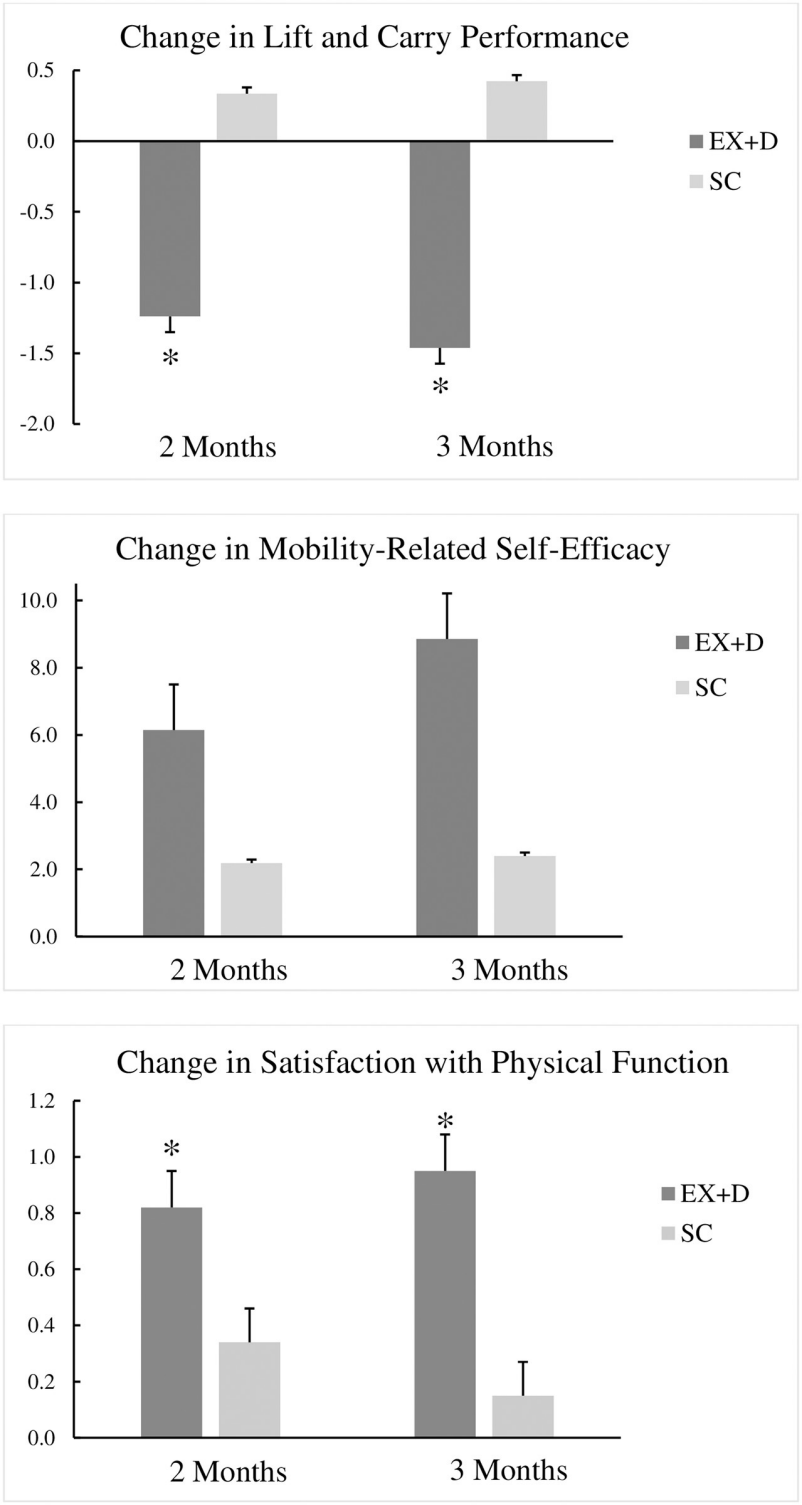
Adjusted mean changes (±SE) in lift and carry performance and social cognitive outcomes.

The unadjusted descriptive statistics for L+C performance, MRSE, and SWF are summarized in Table 1. ANCOVA analyses of baseline-adjusted change scores yielded a significant treatment effect for L+C performance (F[1,28] = 17.90, *p* = 0.006) at 2 and 3 Month follow-up. The EX+D intervention resulted in superior increases in L+C performance at 2 Months (*d* = 1.01; *p* < 0.01) and 3 Months (*d* = 0.95; *p* < 0.01) relative to SC (Fig 2). Although ANCOVA analysis of MRSE revealed a non-significant treatment effect (F[1,28] = 1.94, *p* = 0.18), effect sizes demonstrated that the EX+D intervention resulted in more favorable improvements in MRSE at 2 Months (*d* = 0.38; *p* > 0.05) and 3 Months (*d* = 0.58; *p* > 0.05) relative to SC (Fig 2). Partial correlation analyses controlling for time on ADT and baseline mobility performance yielded inverse correlations revealing that higher levels of task-specific MRSE (*r* = -.66; *p* = 0.001) and SWF (*r* = -.63; *p* = 0.001) were significantly correlated with superior L+C performance at 3 Months. Collectively, these findings revealed the EX+D intervention yielded improvements in L+C performance and MRSE relative to SC and select social cognitive outcomes were associated with more favorable mobility performance at 3 Month follow-up.

**Table 1:** Unadjusted means (SD) for lift and carry performance and task specific mobility-related self-efficacy.

	Intervention Arms	
Variable	EX+D	SC
Lift and Carry Performance		
Baseline	9.00 (3.99)	8.53 (2.12)
2 Month	7.76 (2.56)	8.87 (2.51)
3 Month	7.54 (2.22)	8.96 (2.67)
Mobility-Related Self-Efficacy		
Baseline	86.98 (25.53)	88.02 (13.04)
2 Month	93.13 (12.92)	90.21 (14.33)
3 Month	95.83 (8.11)	90.41 (13.70)
Satisfaction with Physical Function		
Baseline	1.25 (1.13)	0.54 (1.53)
2 Month	1.92 (0.55)	1.03 (1.28)
3 Month	2.04 (0.50)	0.85 (1.02)

## Discussion

The IDEA-P trial is one of the first studies to examine the effects of a combined diet and physical activity lifestyle intervention upon L+C performance and MRSE and explore the extent to which social cognitive constructs are associated with performance of this mobility task. The present findings demonstrated that the theory-based lifestyle intervention, integrating exercise participation, modification of dietary intake, and group-mediated self-regulatory skills counseling, yielded significant improvement in L+C performance and more favorable improvement in MRSE relative to SC patient management. Furthermore, greater task-specific self-efficacy beliefs and satisfaction with function were associated with more favorable post-intervention L+C performance.

To date, relatively few studies have examined the benefits of lifestyle interventions combining exercise and diet in the treatment of PCa patients on ADT [5]. Hence, knowledge of the extent to which there may be substantive links among improvements in social cognitive processes and mobility disability following lifestyle interventions among men undergoing prolonged androgen suppression remains limited [5,6,9]. Therefore, the present findings are novel and demonstrate that key social cognitive constructs, which have been linked with improvements in mobility performance following EX+D interventions in various chronic disease populations [3,4], are also associated with more favorable post-intervention mobility performance in PCa patients on ADT. It is notable that recent evidence demonstrated that improved mobility performance following a weight management intervention was more strongly associated with reductions in total body mass, driven primarily by loss of fat mass, rather than enhanced muscular strength [4]. We previously demonstrated that the EX+D intervention in IDEA-P resulted in significant improvements in mobility, muscular strength, and body composition in PCa patients on ADT and that improvements in adiposity were more strongly related to mobility performance while the lean mass outcomes were more consistently associated with muscular strength [6,13,14]. Collectively, these results underscore the importance of exploring how interactions among change in multiple facets of body composition and social cognitive constructs may contribute to optimizing intervention strategies, personalizing approaches to individuals, and maximizing improvements in mobility performance accompanying lifestyle interventions among PCa patients on ADT in future inquiry.

Although the IDEA-P results are promising, select limitations should be acknowledged. IDEA-P was a single-center pilot trial comprised of a small, relatively homogenous sample thereby limiting the statistical power to detect differences in outcomes of interest and the generalizability of the findings. Accordingly, future multi-center trials targeting more diverse sample of PCa patients are necessary for a more robust evaluation of the observed intervention effects. Additionally, the sample size and brief intervention duration precludes exploring if the social cognitive constructs mediated durable improvements in mobility accompanying the lifestyle intervention, which is a pressing need for future research. Select aspects of the analytical approach must also be considered when interpreting the present findings. For example, whereas an ANCOVA analysis was used to evaluate baseline-adjusted change in the outcomes, generalized estimating equations provide an elegant alternative to analytic approach that should be incorporated in subsequent PCa intervention studies. The intention to treat analysis was conducted using a conservative last value carried forward approach. However, as this approach to the imputation of missing data has well-established limitations and future larger-scale trials incorporating valid, more sophisticated maximum likelihood imputation methods are warranted.

## Conclusions

In summary, the EX+D intervention yielded greater improvement in L+C performance and MBSE relative to SC treatment and select social cognitive outcomes were strongly associated with post-intervention improvements in mobility performance. These findings illustrate the potential value of integrating lifestyle interventions in the supportive care of PCa patients on ADT and suggest social cognitive constructs may have an important role in contributing to the benefits of these interventions for attenuating risk of mobility disability accompanying androgen suppression.

## Supporting information

S1 Checklist (DOCX)

S1 File (DOC)

## References

[1] Bylow K, Mohile SG, Stadler WM, Dale W. Does androgen-deprivation therapy accelerate the development of frailty in older men with prostate cancer?: a conceptual review. Cancer. 2007;110(12): 2604–13. doi: 10.1002/cncr.23084 17960609

[2] Rejeski WJ, Ettinger WH, Schumaker S, James P, Burns R, Elam JT. Assessing performance-related disability in patients with knee osteoarthritis. Osteoarthr Cartil. 1995;3(3): 157–67. doi: 10.1016/s1063-4584(05)80050-0 8581745

[3] Rejeski WJ, Brubaker PH, Goff DC, Bearon LB, McClelland JW, Perri MG, et al. Translating Weight Loss and Physical Activity Programs Into the Community to Preserve Mobility in Older, Obese Adults in Poor Cardiovascular Health. Arch Intern Med. 2011;171(10): 880–886. doi: 10.1001/archinternmed.2010.522 21263080 PMC4425192

[4] Beavers KM, Ambrosius WT, Rejeski WJ, Burdette JH, Walkup MP, Sheedy JL, et al. Effect of Exercise Type During Intentional Weight Loss on Body Composition in Older Adults with Obesity. Obesity (Silver Spring). 2017;25(11): 1823–9. doi: 10.1002/oby.21977 29086504 PMC5678994

[5] Fairman CM, Lucas AR, Grainger E, Clinton SK, Focht BC. The Integration of Exercise and Dietary Lifestyle Interventions into Prostate Cancer Care. In: Platz EA, Berger NA, editors. Energy Balance and Prostate Cancer. Cham, Switzerland: Springer International; 2018. pp. 143–166.

[6] Focht BC, Lucas AR, Grainger E, Simpson C, Fairman CM, Thomas-Ahner JM, et al. Effects of a Group-Mediated Exercise and Dietary Intervention in the Treatment of Prostate Cancer Patients Undergoing Androgen Deprivation Therapy: Results From the IDEA-P Trial. Ann Behav Med. 2018;52(5): 412–428. doi: 10.1093/abm/kax002 29684136 PMC6361261

[7] Bandura A. Sources of self-efficacy. In: Brennan S, Hastings C, editors. Self-Efficacy The Exercise of Control. New York: WH Freeman and Company; 1997. pp. 79–115.

[8] Rejeski WJ, Miller ME, Foy C, Messier SP, Rapp S. Self-efficacy and the progression of functional limitations and self-reported disability in older adults with knee pain. J of Gerontol Soc Sci. 2001;56(5): S261–265. doi: 10.1093/geronb/56.5.s261 11522807

[9] Reboussin BA, Rejeski WJ, Martin KA, Callahan K, Dunn AL, King AC, et al. Correlates of satisfaction with body function and body appearance in middle- and older aged adults: The activity counseling trial (ACT). Psychol Health. 2000;15(2): 239–254.

[10] Schwarzer R. Modeling Health Behavior Change: How to Predict and Modify the Adoption and Maintenance of Health Behaviors. Appl Psychol. 2008;57(1): 1–29.

[11] Schwarzer R, Lippke S, Luszczynska A. Mechanisms of health behavior change in persons with chronic illness or disability: The Health Action Process Approach (HAPA). Rehabil Psychol. 2011;56(3): 161–170. doi: 10.1037/a0024509 21767036

[12] Brawley LR, Rejeski WJ, Gaukstern JE, Ambrosius WT. Social Cognitive Changes Following Weight Loss and Physical Activity Interventions in Obese, Older Adults in Poor Cardiovascular Health. Ann Behav Med. 2012;44(3): 353–364. doi: 10.1007/s12160-012-9390-5 22773225 PMC3593110

[13] Focht BC, Lucas AR, Grainger E, Simpson C, Fairman CM, Thomas-Ahner JM, et al. Effects of a group-mediated cognitive behavioral lifestyle intervention on select social cognitive outcomes in prostate cancer patients undergoing androgen deprivation therapy. Integr Cancer Ther. 2019;18: 1–13. doi: 10.1177/1534735419893764 31838879 PMC6913059

[14] Chaplow ZL, Focht BC, Lucas AR, Grainger E, Simpson C, Buell J, et al. Effects of a lifestyle intervention on body composition in prostate cancer patients on androgen deprivation therapy. JCSM Clin Rep. 2020;5(2): 52–60. 10.1002/crt2.13 PMC9432850 36051892

[15] Rock CL, Doyle C, Demark-Wahnefried W, Meyerhardt J, Courneya KS, Schwartz AL, et al. Nutrition and physical activity guidelines for cancer survivors. CA Cancer J Clin. 2012;62(4): 243–274. doi: 10.3322/caac.21142 22539238

[16] World Cancer Research Fund/American Institute for Cancer Research. Food, Nutrition, Physical Activity and the Prevention of Cancer: A Global Perspective. Washington DC: AICR; 2007. 10.1017/S002966510800712X 18452640

