# Supplementary material for: Social cognitive outcomes are associated with improvements in mobility performance following lifestyle intervention in prostate cancer patients undergoing androgen deprivation therapy
Source: PLoS One. 2022 Jan 27;17(1):e0263136. doi: 10.1371/journal.pone.0263136 (PMC8794107; doi:10.1371/journal.pone.0263136)
Supplement: S1 File — (DOC) [file pone.0263136.s002.doc]

**Local protocol number:** 2012 C0008

**NCI protocol number:** 1R03CA162969-01A1

**Date of protocol activation:** April 16, 2012

**The Intensive Diet and Exercise Adherence Trial – Pilot (IDEA-P):**

**A Feasibility Study of a Lifestyle Intervention in Men**

**Undergoing Androgen Deprivation Therapy**

**Principal Investigator:**

Brian C. Focht, PhD

Assistant Professor, Health and Exercise Science

The Ohio State University

305 W. 17th Avenue

Columbus, OH 43210

ph: 614-292-2165

fax: 614-688-3432

email: [bfocht@ehe.osu.edu](mailto:bfocht@ehe.osu.edu)

**Co-Investigators:**

Steven K. Clinton MD, PhD

Professor, Department of Hematology and Oncology

The Ohio State University

Elizabeth Grainger, PhD, RD

Clinical Research Specialist, Department of Hematology and Oncology

The Ohio State University

Alexander Lucas, MS

Graduate Assistant, Health and Exercise Science

The Ohio State University

**Key Personnel:**

Christi Simpson

Clinical Research Coordinator, Department of Hematology and Oncology

The Ohio State University

**Table of Contents**

**Page**

**1. Study Overview…………………………………………………………… 3**

**2. Background………………………………………………………………… 3**

**3. Objectives………………………………………………………………….. 4**

**4. Significance……………………………………………………………..… 5**

**5. Innovation…………………………………………………………………. 6**

**6. Research Approach and Design………………………………………. 7**

**6.1 Recruitment and Description of Human Subjects………… 7**

**6.2 Inclusion Criteria……………………………………………….. 8**

**6.3 Informed Consent………………………………………………. 8**

**6.4 Minority Representation………………………………………. 8**

**6.5 Methods………………………………………………………….. 8**

**7. Outcome Measures………………………………………………………. 10**

**8. Statistical Considerations…………………………………………….... 11**

**9. Data Safety and Monitoring…………………………………………….. 11**

**10. Risks and Benefits……………………………………………………… 12**

**11. Timeline……………………………………………………….………….. 13**

**12. References………………………………………………………………. 14**

**13. Appendix…………………………………………………………………. 17**

**Study visit schedule**

**Exercise logs**

**Quality of life questionnaires**

**Physical activity questionnaires**

**Food frequency questionnaire (sample)**

**Diet record form**

**1. Study Overview**

Nearly 17% of US cancer survivors are those diagnosed with prostate cancer, a number that now exceeds 2 million men. A significant portion of these men have undergone androgen-deprivation therapy (ADT) as part of a curative approach or are provided with ADT during a prolonged battle with progressive cancer. In 2005, the Institute of Medicine report “*From Cancer Patient to Cancer Survivor: Lost in Transition*” described the inability of primary care teams to manage the effects of cancer therapy (lack of explicit knowledge and guidance) and the inattention from cancer specialists focusing upon disease response. Indeed, we now appreciate that prostate cancer patients endure long and lingering impacts on quality of life (QOL) and health from ADT (low testosterone) as a “trade-off” for more effective cancer cures and prolonged survival. In recent years, research has documented the significant loss of lean muscle mass, reduced muscle strength, and lower bone mineral density, which results in meaningful declines in physical function (PF) and QOL. The aging process may leave older men at higher risk from adverse effects of ADT placing them at greater peril for metabolic syndrome, cardiovascular disease, frailty, and loss of independence.

This protocol addresses a single-blind, 2-arm randomized controlled pilot study of an intensive lifestyle exercise and dietary intervention in PC patients undergoing prolonged ADT. The primary objective of the Intensive Diet and Exercise Adherence-Pilot (IDEA-P) trial is to determine the feasibility and preliminary efficacy of delivering a combined exercise and dietary counseling intervention to prostate cancer patients undergoing ADT. Preliminary data and effect size estimates obtained from this pilot study will be used as the foundation for the design of larger clinical exercise intervention studies in this priority population of prostate cancer patients. A total of 40 to 50 men undergoing ADT who plan to continue ADT for the next 3 months, will be randomly assigned to receive either the lifestyle exercise and dietary intervention (n=20-25) or standard of care disease management education (n=20-25). Assessment of all functional, anthropometric, and QOL outcomes will be obtained at baseline, after completion of the intervention at 2 months, at a follow-up visit 3 months following the baseline assessment, and at a final follow up visit scheduled between 6 and 8 months following baseline (see Figure 2: Study Design).

**2. Background**

Prostate Cancer (PC) affects approximately a quarter of a million men each year and is the second leading cause of cancer mortality in the US (1). Androgen-deprivation therapy (ADT) is the foundation of treatment for men with metastatic PC and is now frequently incorporated into multimodality curative treatment (with radiotherapy and/or surgery) of localized or locally advanced PC (2, 3). However, it has become increasingly evident that men undergoing prolonged ADT endure significant and protracted adverse effects as a “trade-off” for more effective cancer control and increased longevity. In recent years, mounting evidence demonstrates that the catabolic/metabolic effects of ADT result in serious morbidity including loss of lean muscle mass, increased fat mass (e.g., sarcopenic obesity), reduced muscle strength, and lower bone mineral density (4-11). However, it is well established within the weight management literature that modifying both energy expenditure via increased physical activity and energy intake through changes in dietary behavior is integral to successful behavioral weight management interventions. Consistent with this position, emerging evidence from large, randomized controlled trials clearly demonstrates that lifestyle interventions ***combining exercise and dietary*** modification are feasible, efficacious treatment approaches which yield improvements in ***body weight-related outcomes and functional limitations*** that are superior to the effects of exercise or diet alone among older adults with osteoarthritis (OA) and diabetes (32, 38). Primary adverse effects of ADT are increases in body fat/weight and decreases in muscle mass/strength, which in turn, place PC patients at increased risk for functional decline, CVD, and metabolic syndrome. Thus, the synergistic benefits of intensive lifestyle exercise and dietary interventions observed in OA and diabetes patients suggest that this could be an optimal approach to offsetting the adverse effects experienced by PC patients during ADT. Nonetheless, evidence of the feasibility and preliminary efficacy of implementing an intensive lifestyle exercise and dietary intervention during ADT for PC patients remains largely absent. Consequently, the primary objective of the present investigation is to examine the feasibility and preliminary efficacy of an intensive lifestyle intervention combining exercise and dietary modification in PC patients undergoing ADT.

**3. Objectives.** Androgen-deprivation therapy (ADT) is the foundation of treatment for men with metastatic prostate cancer (PC) and is now frequently incorporated into multimodality strategies for the curative treatment of locally advanced PC. Unfortunately, the catabolic effects of ADT result in loss of lean muscle mass, increased fat mass, reduced muscle strength, and lower bone mineral density. In turn, these adverse effects of ADT are linked with functional decline, frailty, and increased risk for cardiovascular disease and metabolic syndrome. Thus, PC patients endure long and lingering impacts on physical function, health status, and quality of life (QOL) that accompany ADT as a “trade-off” for more effective cancer control and extended longevity. Lifestyle modification, including change in exercise and dietary behaviors, may offset, or even reverse, the adverse effects accompanying ADT. However, empirical evidence documenting the feasibility and efficacy of implementing theory-based, personalized exercise and dietary interventions in men undergoing ADT is limited. Given the emerging clinical focus upon improvements in QOL in cancer survivorship, there is a *critical need* to identify the efficacy of intensive lifestyle interventions for countering the risk of functional decline and chronic disease in PC patients undergoing ADT.

Our *overarching research goal* is to conduct studies that inform and solidify the importance of lifestyle exercise and dietary interventions as an integral component of treatment for PC patients undergoing ADT. Our *objectives in this study* are to: 1) determine the feasibility and preliminary efficacy of an intensive lifestyle exercise and dietary intervention in PC patients undergoing ADT and 2) obtain estimates of the effect sizes accompanying changes in clinically relevant disablement process and patient reported outcomes produced by the intensive lifestyle intervention to inform the design of a future definitive randomized controlled lifestyle intervention trial. Our *central hypothesis* is that an intensive lifestyle exercise and dietary intervention will be a feasible, safe, and efficacious approach that produces superior improvements in functional limitations, body composition, and QOL relative to a standard of care treatment approach. This hypothesis is formulated on the basis of strong preliminary data demonstrating that the intensive lifestyle intervention proposed in this investigation yields meaningful improvements in these functional and QOL outcomes among older chronic disease patients at risk for frailty and functional decline. Our *rationale* for this investigation is that its completion would establish the feasibility and initial efficacy of combining exercise and dietary modification in the treatment of men undergoing ADT and provide strong preliminary evidence supporting the systematic implementation of lifestyle interventions in the treatment of PC patients.

To test our central hypothesis and achieve the overall application objectives, we propose the following *Objectives*:

***Objective #1:*** ***To compare the effects of the intensive lifestyle intervention and standard of care treatments on functional limitations (FL), body composition (BC), and QOL in PC patients on ADT.*** Our *working hypothesis* is that the intensive lifestyle exercise and dietary intervention will result in superior improvements in FL, BC, and QOL relative to the standard of care treatment.

***Objective #2:*** ***To determine the feasibility of delivering an intensive lifestyle exercise and dietary intervention to PC patients undergoing ADT.*** Our *working hypothesis* is that the lifestyle intervention will be a safe, well-tolerated intervention that yields acceptable recruitment, adherence, and retention rates and also yields rates of adverse events that do~~es~~ not differ from a standard of care treatment approach.

***Objective #3: To identify the intermediate variables that account for the beneficial effect of the lifestyle intervention on functional limitations and QOL.*** Our *working hypothesis* is that the beneficial effects of the lifestyle intervention on functional limitations and QOL will be indirect, operating through intermediate disablement process model variables including changes in muscle mass and strength, functional performance, and psychosocial outcomes, such as pain, fatigue symptoms, and self-efficacy.

At the completion of this project, we *expect* to have successfully: 1) determined the feasibility and preliminary efficacy of an intensive lifestyle intervention in PC patients undergoing ADT and 2) identified intermediate variables linking change in exercise and dietary behavior to improvements in select functional and QOL outcomes. The primary *positive impact* of our findings would be that they are the first findings supporting the feasibility and efficacy of an evidence-based lifestyle exercise and dietary intervention for offsetting the adverse effects of ADT in PC patients. Findings from the proposed project will also provide the effect size estimates necessary to inform the design of a subsequent R01 application addressing a definitive randomized controlled lifestyle intervention trial targeting men on ADT. The application is also directly responsive to the goal of encouraging investigators to enter and/or transition into the field of behavioral cancer research.

**4. Significance**

**Adverse Effects of ADT Increase Risk of Frailty and Functional Decline.**  Despite the well established therapeutic efficacy of androgen-deprivation therapy (ADT) in the treatment of PC (1), it has become increasingly evident that men on ADT endure lingering adverse effects as a “trade-off” for more effective cancer control and increased longevity. The catabolic effects of ADT result in significant adverse effects including loss of lean muscle mass, increased fat mass, reduced muscle strength, and lower bone mineral density, placing men undergoing ADT at greater risk for functional decline and frailty (2-9). Emerging evidence also suggests that ADT increases risk for cardiovascular disease (CVD) and metabolic syndrome. As prolonged administration of ADT becomes increasingly common, many men will cope with lasting treatment-related side-effects that could meaningfully compromise their physical function and quality of life (QOL). PC is estimated to be the cause of over half a million disability-adjusted life years (10-12). Thus, defining the feasibility and efficacy of innovative interventions that preserve functional abilities and QOL and attenuate risk for chronic disease are primary clinical considerations for PC patients on ADT (2, 7, 13-17).

**A Combined Exercise and Dietary Intervention May Be An Optimal Approach During ADT.** Given the integral role of ADT in PC therapy, there is now a critical need to determine the feasibility and efficacy of supportive care approaches for reducing the adverse effects accompanying androgen suppression. There is growing recognition from researchers and clinicians that ***exercise*** results in improvements in relevant physiologic and patient-reported outcomes across a variety of cancer patients/survivors. (10, 18-30). While few randomized controlled trials have examined the effects of exercise interventions in PC patients undergoing ADT, these findings suggest that exercise consistently results in significant, clinically meaningful improvements in muscular strength, physical function, and QOL (31). However, it is well established within the weight management literature that modifying both energy expenditure via increased physical activity and energy intake through changes in dietary behavior is integral to successful behavioral weight management interventions. Consistent with this position, emerging evidence from large, randomized controlled trials clearly demonstrates that lifestyle interventions ***combining exercise and dietary*** ***modification*** are feasible, efficacious treatment approaches which yield improvements in ***body weight-related outcomes and functional limitations*** that are superior to the effects of exercise or diet alone among older adults with osteoarthritis (OA) and diabetes (32, 38). Primary adverse effects of ADT are increases in body fat/weight and decreases in muscle mass/strength, which, in turn, place PC patients at increased risk for functional decline, CVD, and metabolic syndrome. Thus, the synergistic benefits of intensive lifestyle exercise and dietary interventions observed in OA and diabetes patients suggest that this could be an optimal approach to offsetting the adverse effects experienced by PC patients during ADT. Nonetheless, evidence of the feasibility and preliminary efficacy of implementing an intensive lifestyle exercise and dietary intervention during ADT for PC patients remains largely absent. Determining the feasibility and risk-benefit ratio of implementing a combined exercise and dietary intervention during ADT is a significant research objective that could have meaningful implications for informing best practice clinical approaches to the treatment of PC patients.

**The Intermediate Variables Linking Lifestyle Modification to Improved Physical Function and QOL during ADT are Unknown.** Eliciting improvements in functional limitations and QOL are central considerations in enhancing the treatment of men on ADT. Unfortunately, the pathways through which lifestyle interventions may improve functional limitations and QOL in men on ADT remain unknown. The processes underlying the role of lifestyle modification in attenuating or reversing functional limitations can be best understood through the *Disablement Process Model (DPM)* (42-44), a well-established conceptual framework which proposes that four interrelated components (pathology, impairments, functional limitations, and disability) comprise the primary pathway to functional decline (See Figure 1). Although the DPM is a well-established model for explaining functional decline observed with aging and disease, it has yet to be implemented in intervention studies in PC patients on ADT. Emerging evidence suggests that the beneficial effects of lifestyle interventions on functional limitations and QOL among individuals at risk for frailty and functional decline occur indirectly operating through improvements in intermediate DPM and social cognitive variables such as muscular strength and body composition, functional performance, symptoms of pain and fatigue, and mobility-related self-efficacy beliefs (see Figures 2) (42, 45-50). However, because these pathways have yet to be explored among men on ADT, it is unclear if these same theory-driven intermediate variables account for any improvements in functional limitations or QOL following lifestyle interventions. Accordingly, the proposed project will be the first to identify key intermediate variables in determining the effects of lifestyle interventions on relevant functional limitation and QOL outcomes in men on ADT.

*Figure 1. The Disablement Process Model Applied to PC Patients on ADT.*

**5. Innovation**

**How Does the Application Challenge Current Research or Clinical Practice Paradigms?** The proposed feasibility and preliminary efficacy trial is innovative from both research and clinical practice perspectives. ***From a research perspective,*** an innovative aspect of the proposed study is that it will be the first to determine the safety and efficacy of a theory-driven, evidence-based intensive lifestyle intervention, which has been demonstrated to be safe and efficacious in other populations at risk for functional decline, in men on ADT. Determining the effect sizes accompanying change in the clinically relevant outcomes assessed in this study will also provide the necessary information to inform the design of a subsequent R01 application for a definitive lifestyle exercise and dietary intervention trial in the treatment of PC patients on ADT. The proposed study will also contribute to shifting the direction of exercise-oncology research by being the first to explore the extent to which conceptually-relevant DMP and social cognitive variables, identified in prior lifestyle intervention research, may serve as intermediate variables accounting for the effects of lifestyle interventions on change in functional limitations and QOL in men on ADT. ***From a clinical practice perspective,*** we believe determining the safety and preliminary efficacy of an intensive lifestyle intervention in men on ADT will have meaningful future implications for clinical practice for PC patients. Results of this feasibility study will inform the design of a large randomized controlled lifestyle intervention trial, the findings of which could, in turn, provide the evidence necessary to alter current standard of care practices towards the implementation of exercise and dietary interventions in the routine clinical treatment of PC patients.

**What Novel Approaches or Advantages Over Existing Methodologies Are Implemented?** The theory-driven, evidence-based lifestyle intervention is an innovative approach that makes the proposed study unique from prior or on-going lifestyle interventions targeting men on ADT. Specifically, combining dietary and exercise interventions that include behavioral counseling to facilitate adherence to the behavior modifications adherence is a novel approach from any interventions previously or currently being applied within PC populations. This novel intervention approach has been demonstrated to be feasible and efficacious in producing behavior change and improvements in functional limitations and QOL in prior randomized controlled trials targeting middle-aged and older adults with osteoarthritis (38) and diabetes (32). These same outcomes are clearly relevant to men undergoing ADT, and lifestyle interventions that produce meaningful improvements in these outcomes would unquestionably enhance the efficacy of treatment for these patients.

**Summary and Future Directions.** The absence of evidence addressing the benefits of lifestyle exercise and dietary interventions among men on ADT represents a critical gap in knowledge that undermines the efficacy of clinical and public health practice applied in PC patients. The goal of the proposed study, the **I**ntensive **D**iet and **E**xercise **A**dherence-**P**ilot Trial (**IDEA-P**), is to compare the effects of an intensive lifestyle exercise and dietary intervention with standard of care disease management education. The proposed study addresses a ***significant problem*** for men on ADT and is ***innovative from existing lifestyle intervention research targeting PC patients*** in that it would be the first to: (a) examine the feasibility and efficacy of a theory-driven, evidence-based lifestyle exercise and dietary intervention upon functional and QOL outcomes in men on ADT and (b) explore the extent to which theory-based intermediate variables may act as pathways linking lifestyle change with improvements in functional and QOL outcomes in men on ADT. Findings from the proposed project will provide effect size estimates that will directly inform the design of a subsequent R01 proposal by the present research team for a large, randomized controlled trial addressing the benefits of lifestyle interventions in the treatment of men on ADT. If, as hypothesized, this approach proves beneficial, findings from subsequent definitive lifestyle trials could meaningfully alter current clinical approaches through the systematic integration of exercise and diet as part of the standard of care treatment for PC.

**6. Research Approach and Design**

**Approach.** IDEA-P is a single-blind, 2-arm randomized controlled pilot study designed to determine the feasibility and preliminary efficacy of an intensive lifestyle exercise and dietary intervention in PC patients undergoing prolonged ADT. A total of 40-50 men undergoing ADT will be randomly assigned to receive either the lifestyle exercise and dietary intervention (n=20-25) or standard of care disease management education (n=20-25). Assessment of all functional, anthropometric, and QOL outcomes will be obtained at baseline, after completion of the intervention at 2 months, and at a follow-up visit 3 months following the baseline assessment (see Figure 2).

**
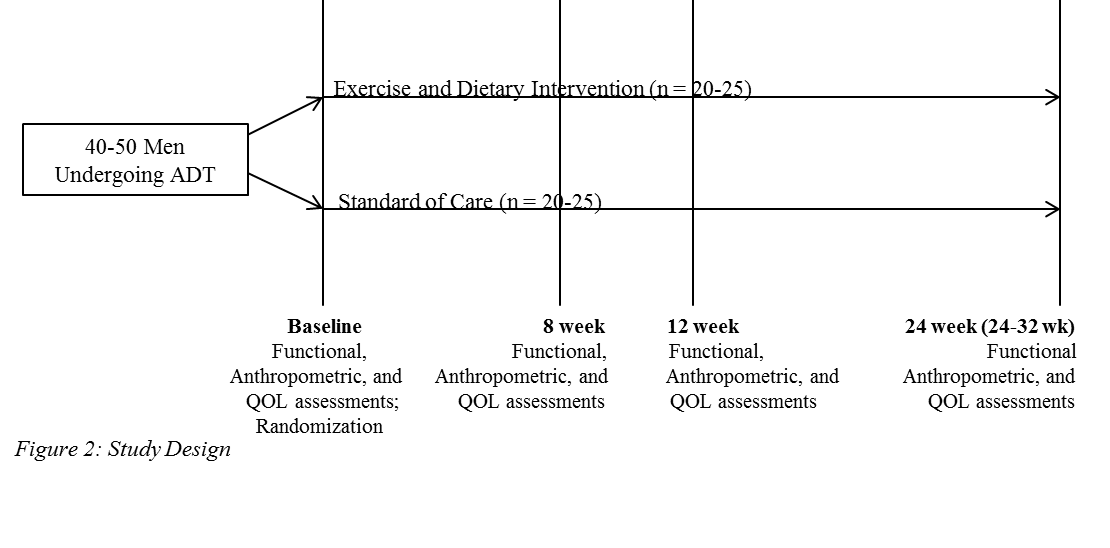
**

**6.1 Recruitment and Description of Human Subjects.** In collaboration with the OSUCCC and the

Genitourinary Oncology Disease Unit, Dr. Clinton will serve as the focal point for recruitment of men for this study. His efforts in this capacity integrate the PC care for the OSUCCC and the JCH. Dr. Clinton also manages a multimodality clinical effort each Wednesday at the JCH, where medical oncologists, radiotherapists, and urologists jointly manage patients. At the present time, well over 1,000 men with PC are referred to JCH each year. Under Dr. Clinton’s direction, the OSU team has led the nation in annual number of local patient accrual to GU clinical trials among the nearly 50 participating institutions of the Cancer and Leukemia Group B (cooperative group) for the last 4 years. In 2009 and 2010 the OSU team has accrued over three times as many GU patients as any other CALGB institution. In addition, Dr. Clinton’s research team has successfully met total accrual via local recruitment of PC patients in several investigator-initiated trials of NIH-NCI-funded diet and nutrition studies.

We can recruit men from the clinics of 4 GU medical oncologists, 5 cancer specific urologists, and 1 GU radiotherapist. Information about the study will also be provided to the Columbus Man to Man PC Support group. These recruitment procedures have resulted in successfully reaching target accrual of both minority and total participant levels in several recent PC clinical trials conducted at the OSUCCC and are evidence of the feasibility of successfully recruiting the target sample. Men will be recruited from Dr. Clinton's clinic and the GU Multimodality Clinic where several hundred men previously treated and cured of PC are monitored annually. To further increase the recruitment rate beyond those patients seen in clinic, we will also mail out a letter of introduction to the study to patients on ADT. We will then follow up the mail out of identified patients with a phone call follow-up. Additional recruitment strategies including flyers, newspaper advertisements, and presentations to local PC support groups will also be used. We are optimistic that we can accrue 3-5 men per month at OSU to the trial. As with all lifestyle interventions, we anticipate some attrition, and even a 20% loss to follow-up would result in a sample sufficient to evaluate the primary aims of interest.

**6.2 Inclusion Criteria**.

• *Diagnosed with PC*: histologically-defined diagnosis of PC based upon providing pathology reports and staging studies.

• *Initiating ADT*: will be undergoing a planned course of at least 3 months of ADT. The ADT is defined as: (a) Surgical castration; (b) GNRH antagonist alone; (c) GNRH antagonist with oral androgen receptor blockade, and (d) GNRH antagonist, oral androgen receptor blockade, and 5-alpha reductase inhibitors. We will not include men with only oral antiandrogen therapy such as 5-alpha reductase inhibitors alone or oral antiandrogens alone, as they do not produce castrate levels of testosterone.

• *Stage*: At enrollment, all men have stage IV disease, which is our major presentation to the medical oncology prostate program. Two groups are typical. The first involves men who usually present for initial diagnosis with minimally metastatic disease and elevated PSA and staging studies with modest nodal enlargement or bone lesions, typically asymptotic. The second group involves men we have been monitoring after local therapy, surgery or radiation, but who have a slow rise in PSA indicating failure of curative therapy. At some point in time asymptomatic metastases are noted on scans, and ADT is offered.

• *Sedentary Lifestyle:* fewer than 60 minutes of participation in structured moderate intensity physical activity each week

• *Health Status*: all participants must be free of active CVD, unstable angina, arrhythmia, or severe systemic disease that would make moderate intensity exercise participation unsafe. Men with prior CVD that have had successful intervention/treatment that are no longer have active CVD and are medically cleared to safely exercise by their physician will be eligible to participate in the study.

• *Consents*: willing to give an informed consent and sign a HIPPA authorization form.

• *Physician Medical Clearance*: all men will have medical clearance to participate in the study from a board certified internist, primary care physician, or cardiologist (for men with ongoing cardiovascular disease) prior to inclusion in the study. All participants’ treating oncologists will also provide consent for participation prior to inclusion in the study.

**6.3 Informed Consent:** Dr. Clinton’s team will obtain informed consent before any assessments are conducted. Potentially interested participants will be identified by Drs. Clinton, Mortazavi, Monk, and Grainger. They will be prescreened and approached in clinic by the study coordinator (Christina Simpson), who, with each patient, will review the consent document, which includes the purpose of the study, study procedures, risks and benefits, confidentiality, and options for withdrawal from the study. Interested participants will then complete the consent form, which will be approved by The Ohio State University Institutional Review Board (IRB). They will be contacted by Dr. Focht, Alex Lucas, and/or Allen Sommer to conduct a telephone screening interview to confirm participants satisfy the eligibility criteria for the study and schedule the baseline assessment visit. The risks of the study protocol are expected to be modest and will be carefully monitored.

**6.4 Minority Representation:** The demographics of the greater Columbus area show a minority population of 17%. Dr. Clinton’s recently completed pre-prostatectomy study (OSU-0137, PI: Dr. Clinton) had a minority recruitment of 18% (6 of 34). Dr. Focht’s recently completed IMPACT-P exercise intervention trial in knee OA patients yielded a minority accrual of approximately 30%. Given these prior accruals in combination with Dr. Clinton’s established success in recruiting minority men undergoing prostate cancer treatment, we anticipate being able to successfully recruit a similar proportion of minority men in the proposed study.

**6.5 Methods for Both Arms (n = 40-50).** Men expressing an interest in the study will complete a brief screening interview to verify eligibility for inclusion in the trial. Men who meet the inclusion criteria can then sign the consent form during the clinic visit. Participants will complete the Dual-energy X-ray Absorptiometry (iDXA) scan anytime within one month of signing consent. The iDXA will be completed using the high definition GE Lunar iDXA. The iDXA machine is located in the gymnasium of Martha Morehouse, the same building at the Oncology Clinic. All other study visits will be conducted at the Recreation and Physical Activity Center on The Ohio State University main campus. **Please see the appendix for a detailed table of study visits**. At the baseline visit, the study will be explained in greater detail to the participant and all questions will be answered. The baseline visit will be comprised of anthropometric assessments, followed by the functional battery, and exercise and nutrition questionnaire assessments (the questionnaires can be completed at home if preferred by the participant) and finally randomization to either the Exercise and Diet Intervention arm or the Standard of Care arm. Participants will be given the option of completing the questionnaire assessments using the online RedCap data acquisition system. The OSU CCTS Research Informatics Services Core will be used as a central location for data processing and management. Vanderbilt University, with collaboration from a consortium of institutional partners (including OSU) and the NIH National Center for Research Resources, has developed a software toolset and workflow methodology for electronic collection and management of research and clinical trial data. REDCap (Research Electronic Data Capture) data collection projects rely on a thorough study-specific data dictionary defined in an iterative self-documenting process by all members of the research team with planning assistance from the CCTS Research Informatics Services Core. As part of the data dictionary development process, individual fields can be denoted as “identifiers”. When exporting a de-identified dataset, these variables are omitted. Additionally, the data export tool also allows for the shifted of dates for a limited data set export. REDCap provides a secure, web-based application that is flexible enough to be used for a variety of types of research, provides an intuitive interface for users to enter data and has real time validation rules (with automated data type and range checks) at the time of entry. It offers easy data manipulation with audit trails and ad hoc reporting functionality for reporting, monitoring and querying patient records, and an automated export mechanism to common statistical packages (SPSS, SAS, Stata, R/S-Plus). REDCap is 21 CRF Part 11 capable. Currently, REDCap installations support electronic signatures by positively identifying the user through a unique username and password combination. The provisioning of accounts and user access to specific database(s) is integrated with the OSU Medical Center LDAP authentication service, and the provisioning of access and specific user rights are managed by CCTS staff. The use of this technology will streamline subject visits and provide the capacity for precoding research data for export and analysis. Participants will be provided verbal and written instructions on how to wear the objective physical activity monitor (LIFECORDER Plus accelerometer) and will wear it for the next 7-days. Monitors will be returned to trial staff via U.S. postal service. Participants will return for three additional assessment visits: two months after baseline, three months after baseline, and between six and eight months after their baseline visits, and assessments identical to that at baseline will be obtained. Assessment of all functional, anthropometric, and QOL outcomes will be obtained at baseline by project staff who are blinded to participants’ treatment arm assignment.

We have preliminary data from our LIVESTRONG pilot study of 17 men on prolonged ADT which demonstrates the proposed assessment battery is safe and well tolerated. All men in this pilot study reported an interest in participating in a lifestyle exercise and diet intervention and 75% reported a preference for programs that involve some combination of supervised and independent exercise. Collectively, these findings demonstrate the successful collaboration among the research team and underscore the potential feasibility of successfully conducting the proposed intervention trial.

**Methods for the Exercise and Diet Intervention Arm (n = 20-25).** The proposed intensive lifestyle intervention, consistent with interventions successfully implemented in prior large-scale lifestyle RCTs (32, 38-41) is a multi-component approach designed to facilitate exercise and dietary behavior change and promote adherence, independent of study staff, to these behavior modifications. The ***exercise component*** involves a combination of aerobic and resistance exercise. The aerobic stimulus consists of 10-30 minutes of exercise performed at a rating of perceived exertion ranging from 11 (Fairly Light) to 14 (Moderately Hard) on the participant’s choice of a treadmill, stationary cycle, or elliptical trainer. The resistance exercise stimulus involves performing 1-3 sets of 8RM-12RM repetitions at a rating of perceived exertion ranging from 12 (Moderately Hard) to 15 (Hard) of 9 different exercises (leg extension, leg curl, chest press, lat pull-down, overhead press, triceps extension, bicep curl, calf raises, and abdominal curl). Exercise duration and intensity will be gradually increased based on individual exercise tolerance and capacity. All exercise sessions will last 1 hour in duration. ***Behavioral activity counseling***, based upon Social Cognitive Theory (45), is also integrated with exercise to promote adherence to the exercise prescription and participant retention. Counseling is delivered via 8 brief (20-min), small group (3-5 participants) sessions conducted once per week immediately following a center-based exercise session during months 1-2. Participants also receive 4 brief (20-min) individualized activity counseling sessions conducted via biweekly phone calls in months 1-3. *The objective of the counseling is to increase self-efficacy for adoption and maintenance of exercise behavior and facilitate the successful transition from supervised to independent center-based exercise participation during the trial.* The behavioral counseling focuses upon the acquisition and practice of self-regulatory skills in conjunction with a continuous problem-solving model of behavior change to empower participants to exert greater control over their behavior, cognitions, and environment. The behavioral counseling is designed to: a) increase health knowledge of the benefits of exercise and dietary change; b) enhance self-efficacy and positive outcome expectancies through the promotion of a series of successful experiences in changing exercise and eating behavior; and c) improve self-regulation of exercise and eating behaviors. The intervention content includes education and counseling efforts involving goal setting, self-monitoring, stimulus control, cognitive restructuring, and barrier problem-solving strategies. To foster the practice/mastery of the newly acquired exercise and behavioral skills and prevent participants from becoming dependent on the expertise of exercise staff to remain physically active, supervised center-based exercise decreases from 2 sessions/week in weeks 1-6 to 1 supervised sessions/week in weeks 7-8 of the intervention. During weeks 7-8, participants have the goal of completing one center-based exercise session independent of study staff supervision during each week. During month 3, participants will have the goal of completing two center-based exercise sessions independent of study staff supervision. Participants will be provided free access to the center-based exercise facility during all its standard operating hours during weeks 7-12. While the facility is supervised by trained fitness staff members during this time, the participants will have no supervisory contact with the study staff during these independent exercise sessions. The ***advantages of the approach integrating counseling and the titration away from staff supervision***are that it helps participants actively apply their developing exercise and behavioral skills to exercise independently while concomitantly providing them access to the study’s exercise facility to facilitate completion of the independent exercise sessions during Weeks 7-12, which also allows us to evaluate uptake of independent exercise adherence in months 2-8. The efficacy of this approach for promoting exercise and dietary behavior change has been demonstrated in prior RCTs on which Dr. Focht has served as principal and co-investigator (38-41).

The ***dietary component*** of the intervention will include 10 (30-min) nutritional counseling sessions with a registered dietitian (Beth Grainger, RD, PhD). The first 8 counseling sessions will be conducted once/week immediately following a center-based exercise session during months 1-2. The 2 remaining sessions are conducted via biweekly phone during Month 3. The specific dietary objectives of IDEA-P will be consistent with the Therapeutic Lifestyle Changes recommended in the Adult Treatment Panel III Report of the National Cholesterol Education Program (62) and the American Institute of Cancer Research (63). The nutrition intervention encourages reductions in portion size and caloric and fat consumption together with a gradual transition from an animal-based diet to a more plant-rich diet while still incorporating animal foods including milk and meat, with an emphasis on monitoring food proportion and portion size. Specific goals of the dietary component include: (a) reduction in energy intake by 500-1000 kcal per day (for men with a BMI of ≥ 27kg/m^2^ only; men with a healthy BMI will be instructed to maintain caloric intake); (b) reduction in total fats to 25-30%, saturated fats to 7%, and protein to 15% of total calories; (c) increase in fruit and vegetable consumption to 5 servings per day; (d) intake of 3 or more servings per day of whole grains per day and a gradual increase to at least 25 grams of dietary fiber per day. The nutrition counseling uses a motivational interviewing approach that has been demonstrated to be an effective approach to promote behavior change in cancer patients (64, 65). The nutrition counseling also builds upon many of the cognitive-behavioral self-management strategies utilized in the exercise intervention including self-monitoring, building self-efficacy, goal setting, and anticipating and overcoming barriers to dietary behavior change. Participants will complete 3-day diet records on two different occasions and these records will be used to guide the counseling sessions and to establish nutrition goals. In each of the diet counseling sessions, a dietitian will review the diet records and weight changes and discuss specific ways to meet the diet goals described above. Each visit will consist of establishing specific and tailored dietary goals for the participant to work on and discussing potential barriers to meeting these goals and developing solutions to these barriers. All the exercise and dietary behavioral counseling conducted during intervention and phone contacts is designed to facilitate the development of behavioral self-regulatory skills needed to successfully adopt and maintain change in exercise and dietary behavior. These contacts will specifically address the following content: self-monitoring (recording/tracking exercise and dietary habits), goal setting, barrier problem-solving, and cognitive restructuring (developing more positive attitude/orientation towards behavior change and its associated challenges) using cognitive behavioral and motivational interviewing approaches. Due to the personalized natures of these intervention approaches, the contacts will not be scripted; rather, they will be tailored~~,~~ to the challenges and/or barriers that individual participants are experiencing. An outline of the topics addressed in the exercise and dietary counseling portion of the intervention is provided below:

- *Self-monitoring*: develop awareness and commitment to tracking/recording of desired exercise and dietary behaviors
- *Goal-setting*: Setting short and long-term, process and outcome-related goals for target exercise and dietary behaviors
- *Stimulus Control*: Development of appropriate behavioral, environmental, and reinforcement strategies that help to facilitate adoption and maintenance of change in exercise and dietary habits (e.g., appropriate exercise pacing, providing oneself with exercise and dietary reminders, identifying appropriate rewards for reaching desired exercise and dietary goals, developing healthier grocery shopping/cooking strategies)
- *Barrier problem-solving*: Use of a social problem-solving approach to identify, practice, and implement self and peer-initiated strategies for overcoming common barriers to exercise and dietary behavior.
- *Cognitive restructuring*: Develop a more positive orientation towards exercise and healthy eating (e.g., monitoring affect during exercise to optimize affect/enjoyment, motivational interviewing to identify positive motives and attitudes for changing exercise and dietary behavior).

**Methods for the Standard of Care Intervention Arm (n = 20-25).** Men randomized to the standard of care arm will receive usual PC treatment as well as educational literature describing the American Institute of Cancer Research dietary and physical activity guidelines. To equate contact between treatment arms to levels consistent with similar, contemporary lifestyle intervention trials (32, 38), 20-min phone contacts delivered by Dr. Clinton’s clinic staff focusing on routine aspects of PC self-management will be conducted biweekly with men in the standard of care arm. As an incentive to promote retention across the trial, men randomized to standard of care will also receive 2 supervised exercise training sessions and dietary counseling sessions following the completion of the 3 month assessment. Men will complete assessments of all outcomes scheduled at baseline and months 2, and 3.

**7. Outcome Measures**

***Functional Battery***. *Functional limitations* will be measured with the abbreviated Late-Life Function and Disability Inventory (LL-FDI) (51). *Functional Performance* will be assessed using 3 valid and reliable timed performance-related mobility tasks: 400 meter walk, stair-climb, and lift and carry task (26, 27, 49, 52). Assessments of *Mobility-Related Self-Efficacy* to complete each functional task will also be completed with the tests.

**Anthropometric and Strength Battery.** *Muscular strength* will be assessed using standardized one-repetition maximum testing protocols for the chest press and leg extension exercises (24, 53). *Body composition* will be assessed using the Bod Pod (Life Measurement Inc, Concord, CA) whole-body air displacement plethysmography system. The Bod Pod has established validity and reliability as an objective method for estimating body composition (54). Another method study staff will use to measure body composition *and bone density* is the GE Lunar iDXA system, which has been proven to provide excellent precision in measurement of total and regional body composition (in addition to bone density) in obese (68) as well as nonobese adults (69). *Body weight* will also be measured to the nearest 0.1 kilogram using a calibrated and certified balance beam scale.

***QOL Battery.*** Global and disease-specific QOL will be assessed using valid and reliable scales including the Satisfaction with Life Scale (7), the SF-36 (55), and the Functional Assessment of Cancer Treatment-Prostate (56) (FACT-P). Pain and fatigue will also be measured with the short-form McGill Pain Questionnaire (57) and the Brief Fatigue Inventory (BFI) (58).

***Behavioral and Feasibility Measures***. Physical activity will be assessed using the *CHAMPS* (50) and *Leisure-Time Exercise Questionnaires* (59). Objectively determined physical activity will also be recorded using the *LIFECORDER Plus Accelerometer* (Suzuken-Kenz, Inc. Nagoya, Japan). Adherence to the exercise prescription will be monitored using exercise logs, upon which participants will record all exercise performed at the center or independently. Exercise-Related Self-Efficacy will be assessed using valid and reliable Exercise Self-Efficacy, Barrier Self-Efficacy, Multi-dimensional, and Self-Regulatory Self-Efficacy scales (57, 60, 61). Dietary assessments will be obtained using the Fred Hutchinson *Food Frequency Questionnaire* and *Three-day diet records*. All behavioral and self-efficacy assessments have established validity and reliability and have been successfully integrated in Dr. Focht and Dr. Clinton’s prior research.

*Feasibility Measures***.** Descriptive statistics for assessments of recruitment rates, intervention adherence, adverse events, and retention rates will be calculated prospectively throughout the trial. Feasibility assessments of participants’ satisfaction with the exercise and dietary intervention will also be completed at the end of the 8-week intervention.

**8. Statistical Considerations.**

Significance tests for the efficacy of the lifestyle intervention on the longitudinally gathered data at 2 and 3 months will be individually standardized by baseline values and will be evaluated using a weighted, repeated-measures analysis of variance statistical model adjusting for the effect of age. The primary aim is to estimate effect sizes for a future application. Although the sample size of 40-50 men does not provide optimal statistical power to detect treatment effects, it is feasible sample to recruit given the time and resources provided by the funding mechanism that, while accounting for a 20% attrition rate, would allow for adequate assessment of the feasibility of the intensive lifestyle intervention and obtain the effect size estimates necessary for the design of a large randomized controlled lifestyle intervention trial. Analyses will be conducted using the intention to treat principle with last value carried forward approach used to account for data missing due to participant attrition. Descriptive statistics for the feasibility measures will be calculated prospectively throughout the trial. Exploratory analyses of the direct and indirect pathways of the intervention effects on change in FL and QOL will be conducted using a structural equation modeling longitudinal panel model approach (AMOS 6.0 software; 66, 67). Panel models are ideal for analysis of hypothesized, theory-based relationships across defined periods of time. Again, while the sample size of 40-50 men is not optimal for panel analyses, it is sufficient to obtain estimates of path coefficients necessary to calculate the sample size in order to design an intervention trial that will be appropriately powered to detect meditational pathways of interest.

**9. Summary of the OSUCCC Data and Safety Monitoring Plan**

The Ohio State University Comprehensive Cancer Center [OSUCCC] places the highest priority on ensuring the safety of patients participating in clinical trials. Every therapeutic interventional trial conducted at the OSUCCC must include a plan for safety and data monitoring. Specific plans may vary based on the degree of risk involved in participation and the size and complexity of the clinical trial. The development of protocol monitoring plans and reporting requirements are dependent upon the study sponsor, nature of the investigational agent, and phase of trial. Trials include studies of cytotoxic and cytostatic agents in treating cancer in an active disease or adjuvant setting, agents targeting biologic endpoints, and interventional studies in cancer control and cancer prevention. Clinical trials sponsored by the NCI Cooperative Group Program [CALGB, GOG, NSABP, CCG, ACoS, RTOG, and SWOG] will be monitored by long-standing and established systems for cooperative group data submission, reporting, review, and monitoring.

All local, investigator-initiated therapeutic clinical trials, such as this proposal, are required to have specific data and safety monitoring plans based on the size and complexity of each trial and the potential risk to patients. Local, investigator-initiated Phase I trials are required to be continuously monitored by the principal investigator of the study with quarterly safety and monitoring reports submitted to the CCC Data and Safety Monitoring Committee [DSMC]. Local, investigator-initiated Phase II protocols will require monthly monitoring by the principal investigator of the study with biannual reports submitted to the DSMC.

Phase I/II studies sponsored by NCI/CTEP will be monitored by the principal investigator with required reporting to NCI/CTEP using the Clinical Trials Monitoring Service [CTMS], the Clinical Data Update

System [CDUS], and/or the Adverse Event Expedited Reporting System [AdEERS]. Due to the difficulty in accessing this data for data and safety monitoring locally, investigators of NCI-supported studies outside the cooperative group mechanism must report to the DSMC as required for local investigator-initiated phase I/II trials. Local, investigator-initiated randomized Phase III clinical trials will be monitored by protocol-specific data and safety monitoring boards [DSMB]. Formal DSMBs will consist of clinical investigators, biostatisticians, clinical trial experts, and lay patient advocates independent of investigators involved in the design and conduct of the trial. Following protocol review and monitoring, all DSMB recommendations and reports will be forwarded to the IRB, DSMB, the CCC Associate Director for Clinical Research, and the principal investigator.

All data and safety monitoring reports for local, investigator-initiated studies will include the number of patients entered, number of patients treated, dose level of agent[s] involved, summary of all adverse events reported to date using CTCAE 3.0 grading, a specific list of adverse events requiring expedited reporting to include ALL serious adverse events [SAEs], and, on an annual basis or as it arises, significant literature reporting developments that may affect the safety of participants or the ethics of the study.

Data safety and monitoring activities for each study will continue until all patients have completed their treatment and all patients are beyond the time point at which study-related adverse events would likely be encountered. Multi-center, limited-institution randomized phase III trials will be held to the same standards as local, investigator-initiated phase III trials and be required to submit formal data and safety monitoring plans including the establishment of an appropriate DSMB prior to activation.

All serious adverse events experienced by patients at the OSUCCC will be immediately reported to the

OSUCCC Clinical Trials Office, the DSMC, the Cancer IRB, and the study sponsor.

Protocol administration and individual patient clinical data will be subject to random audits by the OSUCCC

Office of Protocol Compliance to insure data accuracy and compliance with protocol-directed patient management. In the event that data and safety monitoring by the DSMC results the Associate Director for Clinical Research suspending or terminating a trial, the principal investigator, the OSUCCC Director, the Cancer IRB, the CSRC, and the study sponsor [to include NIH, Department of the Army, pharmaceutical industry sponsors, and other external sponsors] will be notified within 24 hours of such action.

The OSUCCC DSMC will additionally submit an annual report to the OSUCCC Director and OSUCCC

Associate Director for Clinical Research on activities of the past year and make recommendations to improve data and safety monitoring activities. All data and safety monitoring plans, institutional as well as individual protocol-specific, must be reviewed and approved by the OSU Cancer IRB prior to protocol initiation.

**10. Risks and Benefits**

**Risks**: The risks associated with this protocol are expected to be modest. Participants will complete functional and strength testing that has been shown to be of minimal risk of injury or physical discomfort. Men with any medical conditions that would increase the risk of completing these tests or participating in regular, personalized moderate intensity exercise will be excluded from participation. Any physical injury or discomfort will be infrequent, mild, and should not have any long-term effects. To ensure participant safety, all testing and exercise sessions will be conducted under the supervision of an experienced exercise and fitness professionals who is CPR-certified.

As for risk associated with the iDXA scan, additional ionizing radiation has the potential to damage proteins in the body, including DNA, as well as contribute to the subject’s accumulated radiation over time. There are no common adverse effects from bone densitometers and concern is limited to the overall accumulated radiation of each subject.

**Confidentiality:** Patient confidentiality of records will be maintained. Individuals will not be identified by name or by any other personal identifying information in laboratory records, reports, or publications resulting from this study. Thus, risk of disclosure of confidential medical information will be essentially nonexistent. Additionally, all data will be number coded and stored in a locked research office that can only be accessed by the research staff. These procedures have been successfully used to protect confidentiality in both Dr. Focht and Dr. Clinton’s prior completed research.

**Benefits**: The benefits to the participant are significant. Men enrolled in the diet and exercise arm may improve their fitness over the course of the three month study. Men may feel more energetic, confident and activities of daily living may be easier. Additionally, the combination of diet and exercise counseling may facilitate weight loss in men who are overweight which can result in improved health and self image. All men who participate in this clinical trial will potentially have feelings of self-satisfaction for having contributed toward scientific understanding of prostate cancer treatment. Additionally, we will provide a $25 cash incentive for the completion of each major assessment visit (baseline, month 2, month 3, and month 6-8) for a total of $100 for study completion.

**11. Project Timeline**

Database development and personnel training will occur in the first 2 months of Year 1. Participant recruitment, with a target accrual of 3-5 participants per month, will begin month 4 of Year 1 and continue through month 7 of year 2. The intervention data collection will begin in Month 6 of Year 1 and end in month 10 of Year 2. The remaining months of Year 2 will be used for data analyses, presentations, and manuscript preparation and provide flexibility in the event of unexpected barriers during data collection.

Table 1. Timeline for performance of major study activities.

|  | Year 1 | Year 2 |
| --- | --- | --- |
| Activity | 1 2 3 4 5 6 7 8 9 10 11 12 | 1 2 3 4 5 6 7 8 9 10 11 12 |
| Database Development & Training | X --------- |  |
| Recruitment | X -------------------------------- | ------------------------- |
| Intervention & Follow-Up Data Collection | X-------------------------- | ------------------------------------- |
| Analyses/Presentations |  | X------------- |

**12. References**

**APPENDIX**

**Study Schedule for Diet and Exercise Intervention Group**

|  |  | | **Month 1** | | | | **Month 2** | | | | **Month 3** | | | | **Months 6-8** |
| --- | --- | --- | --- | --- | --- | --- | --- | --- | --- | --- | --- | --- | --- | --- | --- |
|  | **Oncology**  **Clinic**  **Visit** | **Baseline**  **and Randomization** | **Week**  **1** | **Week**  **2** | **Week**  **3** | **Week**  **4** | **Week**  **5** | **Week**  **6** | **Week**  **7** | **Week**  **8** | **Week**  **9** | **Week**  **10** | **Week**  **11** | **Week**  **12** | **Weeks 24-32** |
| **Sign Consent** | **x** |  |  |  |  |  |  |  |  |  |  |  |  |  |  |
| **iDXA Scan** | **x** |  |  |  |  |  |  |  |  |  |  |  |  | **x** |  |
| **Physical Assessment**  **(*functional and strength battery, anthropometric measures)*** |  | **x** |  |  |  |  |  |  |  | **x** |  |  |  | **x** | **x** |
| **Supervised Exercise Sessions** |  |  | **2x** | **2x** | **2x** | **2x** | **2x** | **2x** | **x** | **x** | Frequency determined by participant | | | |  |
| **Behavioral Counseling** |  |  | **x** | **x** | **x** | **x** | **x** | **x** | **x** | **x** |  |  |  |  |  |
| **Behavioral Follow up by telephone** |  |  |  | **x** |  | **x** |  | **x** |  | **x** |  | **x** |  | **x** |  |
| **Nutrition Counseling** |  |  | **x** | **x** | **x** | **x** | **x** | **x** | **x** | **x** |  |  |  |  |  |
| **Nutrition follow up by telephone** |  |  |  |  |  |  |  |  |  |  | **x** |  | **x** |  |  |
| **Quality of Life Questionnaires** |  | **x** |  |  |  |  |  |  |  | **x** |  |  |  | **x** | **x** |
| **Behavioral & Feasibility Questionnaires** |  | **x** |  |  |  |  |  |  |  | **x** |  |  |  | **x** | **x** |
| **National Institutes of Health FFQ and Fruit & Vegetable Screener** |  | **x** |  |  |  |  |  |  |  |  |  |  |  | **x** | **x** |
| **Three Day Diet Records** |  | **x** |  |  |  |  |  |  |  |  |  |  |  | **x** | **x** |

**Study Schedule for Standard-of-Care Group**

|  |  | |  | **Month 1** | | | | **Month 2** | | | | **Month 3** | | | | **Months 6-8** |
| --- | --- | --- | --- | --- | --- | --- | --- | --- | --- | --- | --- | --- | --- | --- | --- | --- |
|  | **Oncology**  **Clinic Visit** | | **Baseline and Randomization** | **Week**  **1** | **Week**  **2** | **Week**  **3** | **Week**  **4** | **Week**  **5** | **Week**  **6** | **Week**  **7** | **Week**  **8** | **Week**  **9** | **Week**  **10** | **Week**  **11** | **Week**  **12** | **Weeks 24-32** |
| **Sign Consent** | **x** | |  |  |  |  |  |  |  |  |  |  |  |  |  |  |
| **iDXA Scan** | **x** | |  |  |  |  |  |  |  |  |  |  |  |  | **x** |  |
| **Physical Assessment**  **(*functional and strength battery, anthropometric measures)*** |  | | **x** |  |  |  |  |  |  |  | **x** |  |  |  | **x** | **x** |
| **Phone Contact regarding PCa care** |  | |  |  | **x** |  | **x** |  | **x** |  | **x** |  | **x** |  | **x** |  |
| **Quality of Life Questionnaires** |  | | **x** |  |  |  |  |  |  |  | **x** |  |  |  | **x** | **x** |
| **Behavioral & Feasibility Questionnaires** |  | | **x** |  |  |  |  |  |  |  | **x** |  |  |  | **x** | **x** |
| **National Institutes of Health FFQ and Fruit & Vegetable Screener** |  | | **x** |  |  |  |  |  |  |  |  |  |  |  | **x** | **x** |
| **Three Day Diet Records** | |  | **x** |  |  |  |  |  |  |  |  |  |  |  | **x** | **x** |
